# Modeling and tracking Covid-19 cases using Big Data analytics on HPCC system platform

**DOI:** 10.1186/s40537-021-00423-z

**Published:** 2021-02-15

**Authors:** Flavio Villanustre, Arjuna Chala, Roger Dev, Lili Xu, Jesse Shaw LexisNexis, Borko Furht, Taghi Khoshgoftaar

**Affiliations:** 1 LexisNexis Risk Solutions, Atlanta, Georgia; 2 Florida Atlantic University, Boca Raton, FL USA

**Keywords:** Modeling Corona spread, SARS-Cov-2, Covid-19, Spreading indicators, Big Data, HPCC system

## Abstract

This project is funded by the US National Science Foundation (NSF) through their NSF RAPID program under the title “Modeling Corona Spread Using Big Data Analytics.” The project is a joint effort between the Department of Computer & Electrical Engineering and Computer Science at FAU and a research group from LexisNexis Risk Solutions.

The novel coronavirus Covid-19 originated in China in early December 2019 and has rapidly spread to many countries around the globe, with the number of confirmed cases increasing every day. Covid-19 is officially a pandemic. It is a novel infection with serious clinical manifestations, including death, and it has reached at least 124 countries and territories. Although the ultimate course and impact of Covid-19 are uncertain, it is not merely possible but likely that the disease will produce enough severe illness to overwhelm the worldwide health care infrastructure. Emerging viral pandemics can place extraordinary and sustained demands on public health and health systems and on providers of essential community services.

Modeling the Covid-19 pandemic spread is challenging. But there are data that can be used to project resource demands. Estimates of the reproductive number (R) of SARS-CoV-2 show that at the beginning of the epidemic, each infected person spreads the virus to at least two others, on average (Emanuel et al. in N Engl J Med. 2020, Livingston and Bucher in JAMA 323(14):1335, 2020). A conservatively low estimate is that 5 % of the population could become infected within 3 months. Preliminary data from China and Italy regarding the distribution of case severity and fatality vary widely (Wu and McGoogan in JAMA 323(13):1239–42, 2020). A recent large-scale analysis from China suggests that 80 % of those infected either are asymptomatic or have mild symptoms; a finding that implies that demand for advanced medical services might apply to only 20 % of the total infected. Of patients infected with Covid-19, about 15 % have severe illness and 5 % have critical illness (Emanuel et al. in N Engl J Med. 2020). Overall, mortality ranges from 0.25 % to as high as 3.0 % (Emanuel et al. in N Engl J Med. 2020, Wilson et al. in Emerg Infect Dis 26(6):1339, 2020). Case fatality rates are much higher for vulnerable populations, such as persons over the age of 80 years (> 14 %) and those with coexisting conditions (10 % for those with cardiovascular disease and 7 % for those with diabetes) (Emanuel et al. in N Engl J Med. 2020). Overall, Covid-19 is substantially deadlier than seasonal influenza, which has a mortality of roughly 0.1 %.

Public health efforts depend heavily on predicting how diseases such as those caused by Covid-19 spread across the globe. During the early days of a new outbreak, when reliable data are still scarce, researchers turn to mathematical models that can predict where people who could be infected are going and how likely they are to bring the disease with them. These computational methods use known statistical equations that calculate the probability of individuals transmitting the illness. Modern computational power allows these models to quickly incorporate multiple inputs, such as a given disease’s ability to pass from person to person and the movement patterns of potentially infected people traveling by air and land. This process sometimes involves making assumptions about unknown factors, such as an individual’s exact travel pattern. By plugging in different possible versions of each input, however, researchers can update the models as new information becomes available and compare their results to observed patterns for the illness.

In this paper we describe the development a model of Corona spread by using innovative big data analytics techniques and tools. We leveraged our experience from research in modeling Ebola spread (Shaw et al. Modeling Ebola Spread and Using HPCC/KEL System. In: Big Data Technologies and Applications 2016 (pp. 347-385). Springer, Cham) to successfully model Corona spread, we will obtain new results, and help in reducing the number of Corona patients. We closely collaborated with LexisNexis, which is a leading US data analytics company and a member of our NSF I/UCRC for Advanced Knowledge Enablement.

The lack of a comprehensive view and informative analysis of the status of the pandemic can also cause panic and instability within society. Our work proposes the HPCC Systems Covid-19 tracker, which provides a multi-level view of the pandemic with the informative virus spreading indicators in a timely manner. The system embeds a classical epidemiological model known as SIR and spreading indicators based on causal model. The data solution of the tracker is built on top of the Big Data processing platform HPCC Systems, from ingesting and tracking of various data sources to fast delivery of the data to the public. The HPCC Systems Covid-19 tracker presents the Covid-19 data on a daily, weekly, and cumulative basis up to global-level and down to the county-level. It also provides statistical analysis for each level such as new cases per 100,000 population. The primary analysis such as Contagion Risk and Infection State is based on causal model with a seven-day sliding window. Our work has been released as a publicly available website to the world and attracted a great volume of traffic. The project is open-sourced and available on GitHub. The system was developed on the LexisNexis HPCC Systems, which is briefly described in the paper.

## Modeling Corona spread patterns

**Fig. 1:**
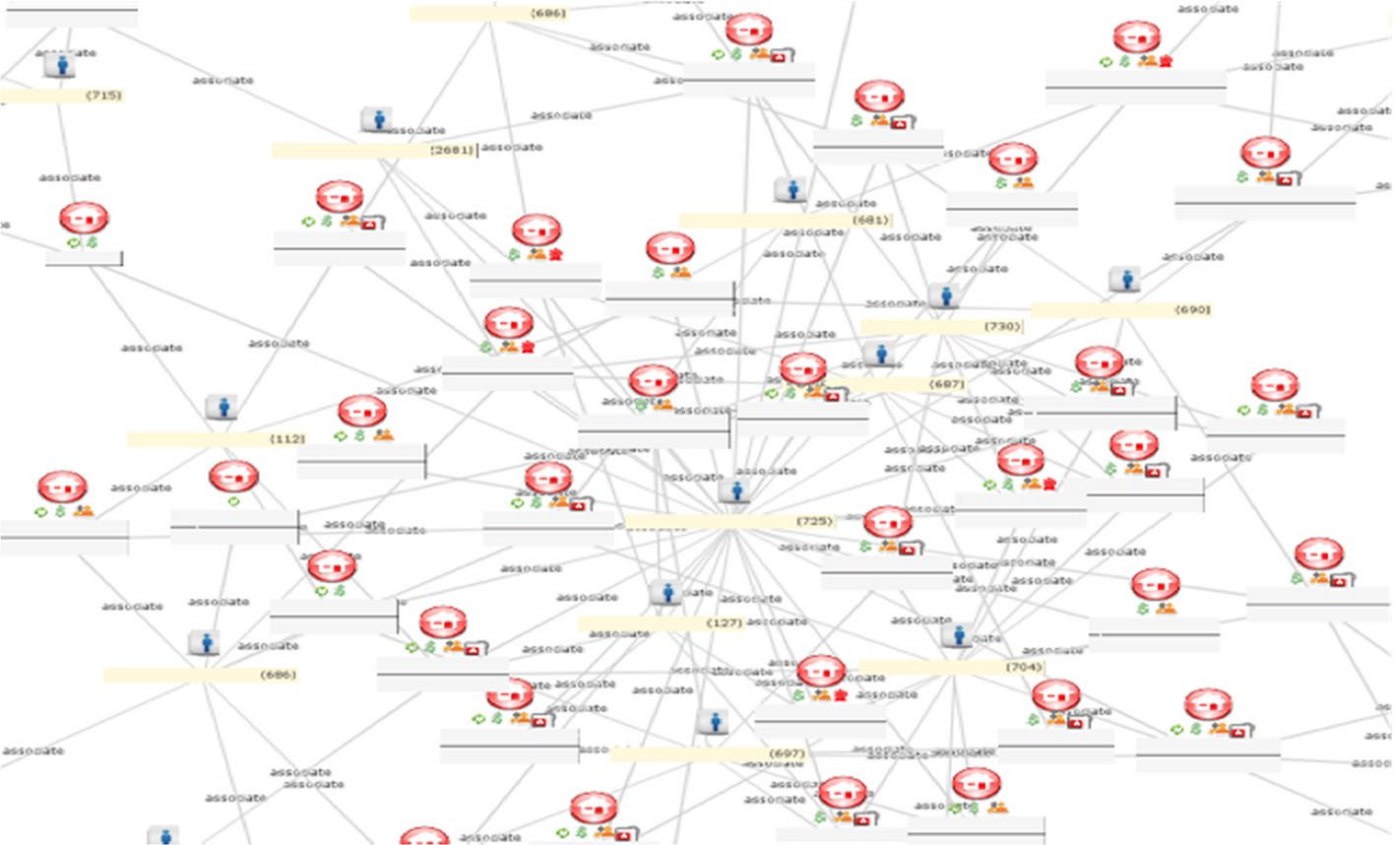
A network of individuals associated by personal relationships

Infectious disease spread across populations usually follows well-defined patterns determined by the transmission mechanisms that the pathogen can use and the network of relationships that the pathogenic agent can follow to spread throughout a community. For those contagious diseases where the transmission can be direct from person to person and airborne, even short and transient exposures to microscopic particles in the air, in enclosed areas where victims breathe, can be sufficient to propagate the disease. LexisNexis, as indicated earlier, is committed to providing a large amount of data about the relationship of the people in US, as illustrated in Fig. 1.

Using Big Data analytic techniques, data about underlying personal relationships, health center locations and the known mechanisms for spread of the Corona virus, this research will study computational models to predict the spread of this disease utilizing both, forward simulation from a given patient and the propagation of the infection into the community and backward simulation, tracing a number of verified infections to a possible patient “zero” [1–5].

**Fig. 2:**
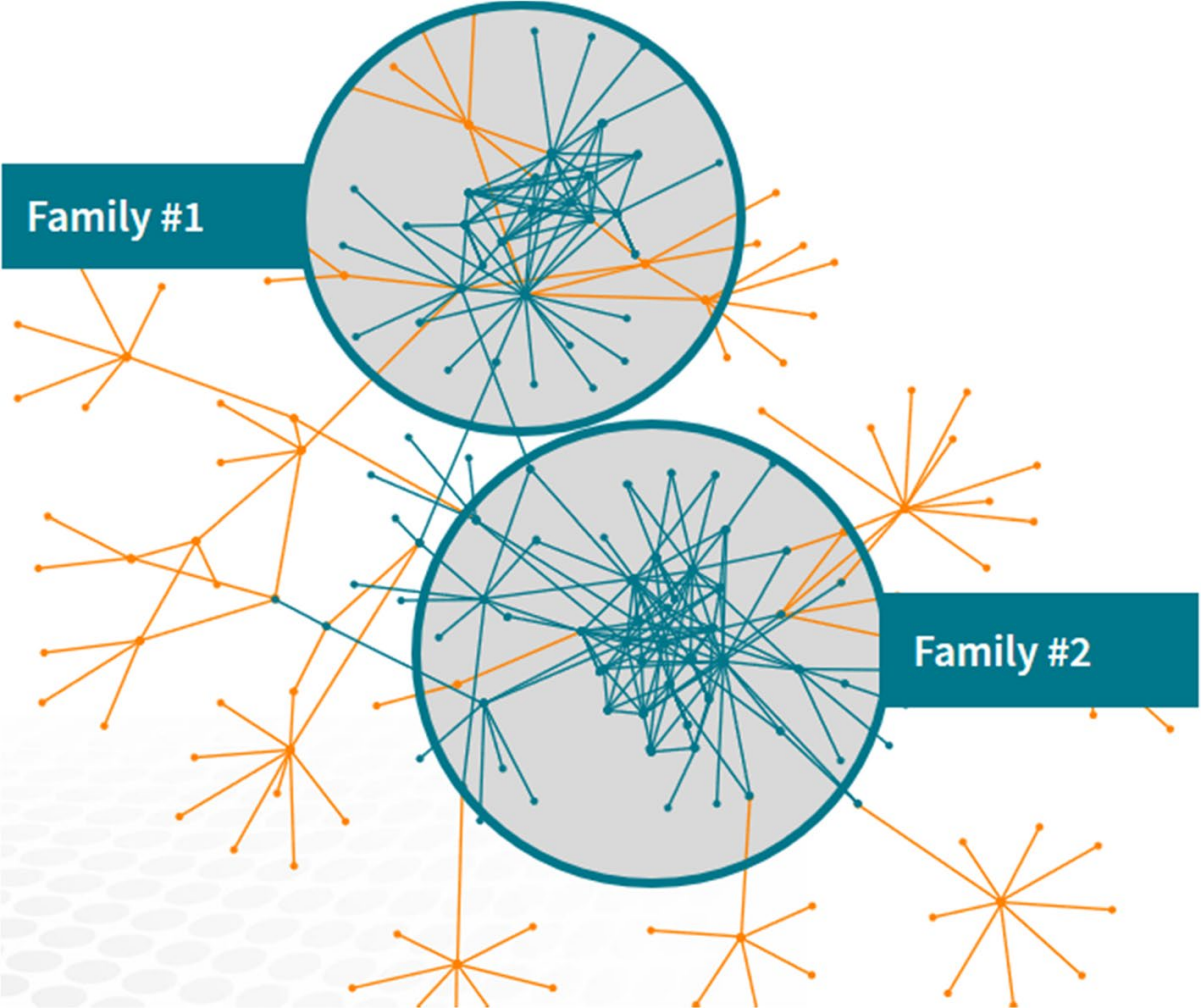
Identifying families and tightly connected social groups

It will also be possible to create alternative models that could account for mutations in the virus that could change the mechanism used for spread, taking seasonal migrations into account and evaluating the impact on the spreading by mitigating factors such as quarantine, change of habits, etc. Figure 2 illustrates how the system will identify and visualize families and tightly connected social groups who have connections with a Corona patent. We will use big data analytics tools for combining available information of an infectious disease process, transforming such information into practical knowledge, and detecting and predicting disease epidemics. In this project, we propose to investigate compartmental models and dynamic diffusion models in mining spread patterns of Corona.

### Compartmental models

Mathematical compartmental models have been successfully applied to predict the behavior of disease outbreaks in many studies [6, 7]. These models aim to understand the dynamics of a disease propagation process and focus on partitioning the population into several health states. For example, in the classical SIR model, three compartments are labeled as susceptible (S), infectious (I), and immune (R, for recovered). The model estimates the number of people getting infected due to direct contact with an infected individual at a certain time. Additional compartments such as exposed (E, representing an individual in incubation period), hospitalized (H), and funeral (F) can also be added into the model [8]. In these models, a basic reproductive rate is usually defined to represent new cases expected to be produced by an infectious individual. When control interventions are taken, the rate should decline. In this scenario, exponential adjustments will be introduced to make the models adaptable to continuous situation changes caused by various control mechanisms [9].

### Dynamic diffusion models

With information from multiple sources indicating infected individuals and their personal relationships and social groups, dynamic graphs can be created [10, 11] and predictive diffusion models can be used to study key issues of Corona epidemics, e.g., location, time and number of expected new cases. Two fundamental diffusion models are Independent Cascade Model (IC) and Linear Threshold Model (LT), both of which follow an iterative diffusion process, and in each iteration uninfected nodes will be infected by their infectious neighbors with certain probabilities [11]. Based on fundamental models, advanced propagation models can be built to estimate an influence function by examining past and newly infected nodes and predict subsequent infections [12, 13]. Other graph-based data mining and machine learning techniques, including continuous-time Markov process analysis [6], label propagation [14], active learning [15, 16], and mixture models [17] could also be explored to create realistic computational models for the spread prediction.

## Risk score approach in modeling and predicting Corona Spread

Modern disease compartmental models are developed to the point where the most significant factors controlling propagation make up components in the name. Since propagation varies from disease to disease, this model naming convention can loosely serve as a classification for disease type which represents simple diseases: from the common cold or influenza (SIS) to pathogens more complex in nature such as Corona (SEIR). Compartmental models produce efficient estimates for pathogen prevalence and duration, and this insight is vital in stopping highly contagious diseases like Corona. This infection period would also be marked with an asymptomatic characteristic, meaning a host is infected but no symptoms are presenting (SEIaR).

Because of its protracted asymptomatic period and virulence, Corona can spread quickly unless strategic precautions are taken, including re-examining the compartmental model to account for newly observed spread characteristics.

### Beyond compartmental modeling

The basis for compartmental models is making assumptions about social networks or graphs. Common assumptions can include: number of individuals, infection probability, incubation period, infected recovery time, etc. These phenomenological assumptions limit the scope of the model while preserving the most realistic aspects of it, but some model dimension assumptions are necessary because actual social network data does not exist. In the era of “big data” this is quickly changing.

Corporations across the globe are becoming experts at the collection of transactional data. While some of the data captured is specifically to enhance automated decision-making systems, the majority of data collected is still in a raw, unleveraged form, making knowledge extraction the next field to experience an explosion of growth. On the forefront of knowledge extraction, LexisNexis produced the RELX Social Graph consisting of over 4 billion relationships built from applied identity analytics on a 4 petabyte core of content [5].

### Physical and social graphs

**Fig. 3:**
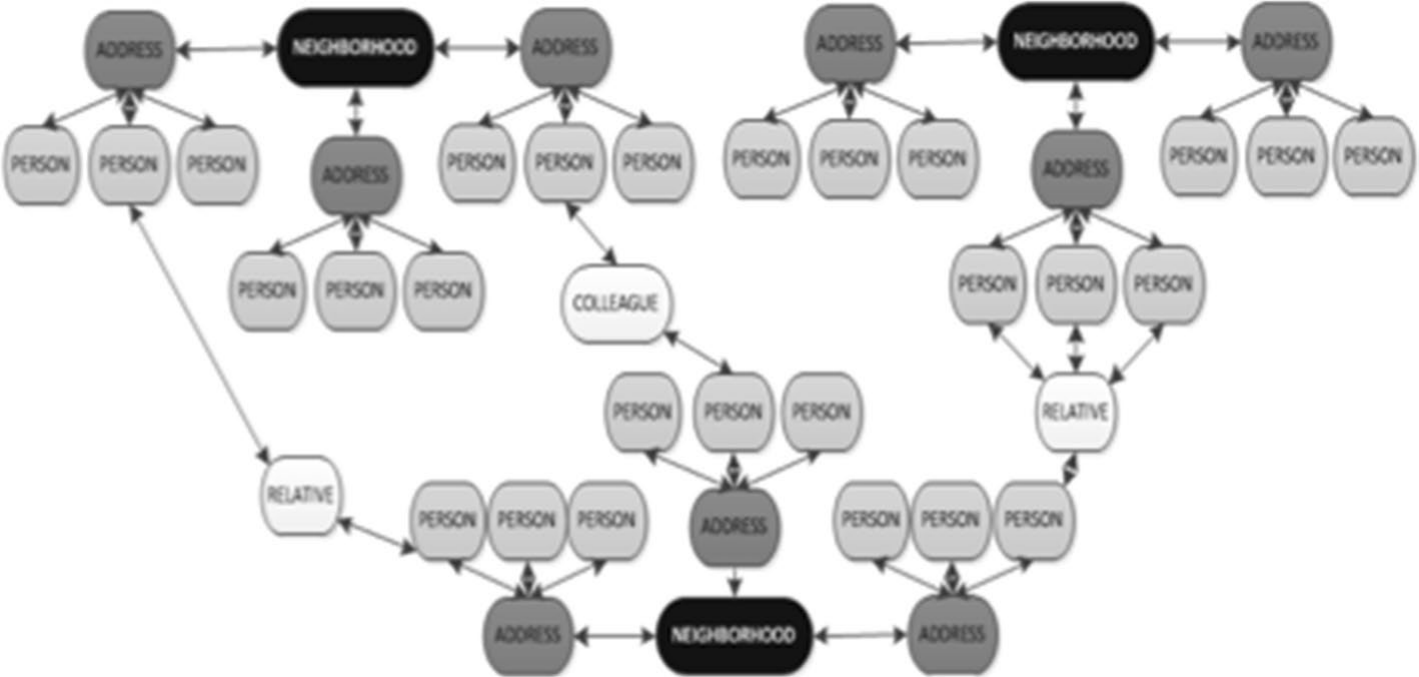
A physical network is constructed by proximity resulting in a ‘nearest neighbor’ linking

Unlike user-curated social graphs such as Facebook, the RELX graph coalesces as people experience life events. Sharing employers, addresses, insurance policies, and vehicle or property ownership are examples of the life events linking two people together. Applied graph analytics appends useful measures to help describe the quality of clusters. For the purposes of measuring the risk of a cluster contracting/propagating a disease, physical proximity of nodes (regardless of social connection) also plays a critical role. The physical proximity calculation between nodes is a simple distance calculation for each of the subject’s most current address. A traditional social network does not imply a physical network, but a physical network may imply the subset of a social network. A physical network is constructed by proximity resulting in a ‘nearest neighbor’ linking, as illustrated in Fig. 3. Proximity, however, does not guarantee contact, and therefore, a combination of proximity and social linking should be considered.

### Graph knowledge extraction

Tools such as Gephi, NodeXL, or SVAT offer intuitive visual searches and a basic set of network measures, but to move beyond superficial graph descriptors to real-world application a different approach must be taken. Similar in nature to Neo4j, the RELX Knowledge Engineering Language (KEL) provides the ability to blend massive graph databases (billions of records) and derive dimensions beyond simple relational properties. As mentioned earlier, performing a distance calculation between nodes creates an additional edge weight distance. KEL can not only calculate the most recent difference in addresses, but also a chronology of addresses, providing metrics such as cluster mobility, average move distance, physical cluster expansion/contraction, address density, occupant density, and many others.

Since the SEIR model places emphasis on physical proximity and social cohesivity, can the spread of information about the disease outpace the spread of the disease, thereby slowing its progression? Is disease transmission highest when a cluster is highly proximal, but non- cohesive socially? Do friendly people keep us safe from diseases like Corona by serving as connectors helping to propagate awareness between disparate social groups faster than the disease can spread? Research conducted by Damon Centola from the University of Pennsylvania titled “*The Social Origins of Networks and Diffusion*” suggests the diffusion of ideas is as sensitive to the homogeneity of the network. Idea diffusion requires a network to be “just right”: moderately homogenous and moderately connected. A highly homogenous or under-connected graph population results in poor idea propagation.

Applying this idea on the national scoped RELX Graph: “*Which clusters have: the largest first degree count, the lowest average degree (cohesivity measure), the highest neighbor count, the highest colleague count, haven’t moved in 5 years, and live in an area where there are few single family dwellings and car ownership is 1:10?”* These people are highly connected, live in a metro area, rely on public transportation, commute to work, and know their neighbors.

The nodes identified by this filter are key influencers and could be leveraged to proactively slow the propagation of a physically communicable disease like Corona, potentially limiting the exposure to health care workers and their networks. Future refinements could include the incorporation of a health care worker flag or proximity to a health care facility; homogeneity dimensions such as: political affiliation, economic trajectory, or migration velocity; or proximity to public transportation hubs: bus and train stations or airports.

Graph Propagation  Points of intervention can also be identified by simulating the propagation of a disease based on SEIR model dimensions as edge characteristics. The node selected for intervention would be the first non-exposed node found on the most infectious, shortest path. The most infectious, shortest path is defined as: the shortest path in a sub-graph through which the number of first degree nodes is maximized. KEL does not have native graph traversal rules distinguishing between a *walk* and a *path*; however, KEL does allow for the creation of such rules. To control backtracking, or double counting nodes as nth degree relatives, the GLOBAL primitive is used.

Expanding the rules out to eight degrees exceeds the largest inter-cluster diameter found in the RELX graph. Applying these rules to a sample data set produced the desired results. Graph traversal rules identifies the root, sink, intermediate nodes, total distance traveled, the number of unique first degree nodes encountered along the path, the total path length, and percent of nodes encountered during traversal. Shortest path does not guarantee most infectious.

## LexisNexis HPCC systems platform

**Fig. 4:**
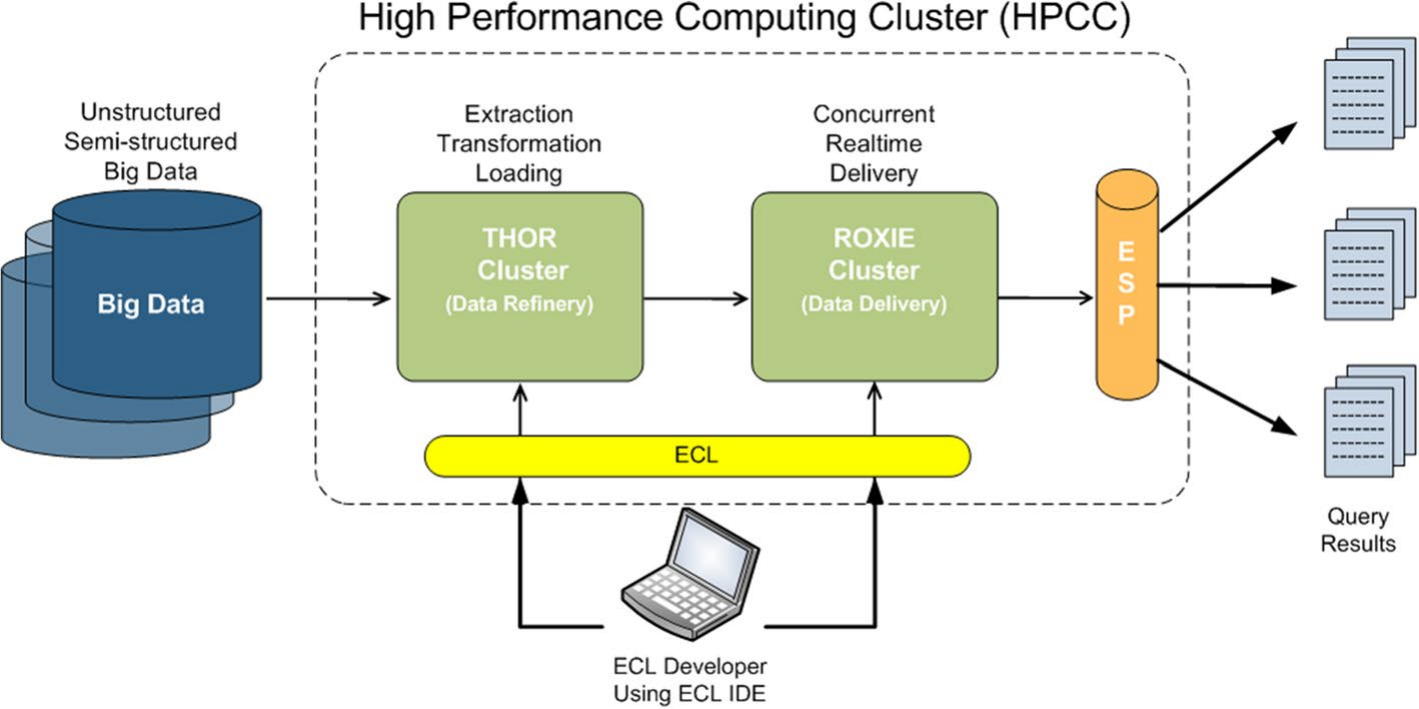
Architecture of the Open Source HPCC Systems

This research leveraged the Open Source HPCC Systems Big Data technology platform originally developed at LexisNexis (shown in Fig. 4).

The HPCC platform incorporates a software architecture implemented on commodity computing clusters to provide high-performance, data-parallel processing for applications utilizing big data. The HPCC platform includes system configurations to support both parallel batch data processing (Thor) and high-performance online query applications using indexed data files (Roxie). The HPCC platform also includes a data-centric declarative programming language for parallel data processing called ECL. The HPCC Systems platform can also efficiently process time-series data, making it an ideal tool to process and analyze data that models evolution over time of one or many multiple states. In addition to the technology platform, LexisNexis’ data on personal relationships and associations in the United States will be used in developing realistic spread models for the disease, in combination with location data for public health services.

## Developing Covid-19 tracker

The Covid-19 crisis moves at a very quick pace. Changes in social behavior can result in rapid changes to the overall picture. Monthly analysis would be obsolete by the time the analysis was available. Daily results are too noisy to be useful for the decision-making. We therefore chose weekly analysis as the optimal time frame to understand the situation. While we present the data on a daily, weekly, and cumulative basis, the primary analysis of the current situation is based on a sliding seven-day window.

We start by trying to understand the spread rate of the virus in a particular population (i.e. location). Epidemiology uses an effective reproductive growth rate known as “R” to represent the speed of spread. R defines the number of people a single individual is likely to infect over the course of their infection. Infections spread exponentially, and R represents the base of the exponent. An R value equal to 1.0 would represent a steady state in the number of active infections—each person would infect one other person, so the overall infection rate (number of active infections) would not change. An R value greater than 1 means that the infection is growing among the population, while an R value below 1 indicates that the infection is subsiding. With an R of 2.0, an infection would double the number of new cases every ten days or so. A higher R value means a faster the infection rate. Likewise, an R of 0.5 would halve the number of new cases every ten days.

We cannot observe R in practice, so we try to approximate it from the number of confirmed cases and the number of deaths due to the virus. The approximation is based on confirmed cases that we define as Case Growth (cR). We define the approximation based on deaths as Mortality Growth (mR). These numbers do not perfectly reflect R, but they are the best available approximations. Case Growth cR is biased by changing availability and policies around testing. If we had randomized testing, we could better approximate R. If we only test hospitalized patients, then cR will understate R. If the testing policy is shifting, then cR may either under or overstate R. Mortality Growth mR, on the other hand, is a more objective indicator. It is less affected by policy, but may be biased by changes in medical care, such as improved treatments over time. mR also lags cR, so it is not as timely an indicator. By combining mR and cR, we get a better overall approximation of R.

By approximating R, we can quickly assess the situation in a given location. As an infection spreads within a location, one of two situations typically arises:

- The infected people will be quarantined and their contacts traced and also quarantined. If this is successful, the infection would be contained, and R will quickly decrease.
- The containment fails, either due to late detection, failure to trace all contacts, or due to insufficient resources to enact the containment. In this case, the infection will spread uncontrolled until social behavior (e.g. social distancing) causes it to be controlled. This process is referred as “Mitigation.”

By following changes in R, we can quickly assess how the infection is responding to Containment or Mitigation. In the early emergent stages of the infection, we commonly see R values greater than 3, which indicates a very fast exponential infection growth. As the infection is contained or social distancing is deployed, R quickly falls to between 1 and 2, which can still be very rapid growth. At R = 2, the cases will double every 10 days. As the case growth increases, people tend to become more and more careful until R falls below 1. At this point the active infections stop increasing and gradually begin to decrease. We expect that this will tend to make people less careful, and we expect to see oscillation above and below 1. If social distancing can be maintained for a longer period, then the infection can be ultimately re-contained.

## Models and metrics

We use an evolving model of the cause and effect relationships between observed and unobserved (latent) variables to inform the definition and interpretation of metrics. This model lets us visualize the ways in which measurements are confounded by hidden variables, and possible ways to de-confound the meanings. J. Pearl [19] has demonstrated that people are extremely good at building causal models. It may be that the human mind is largely a causality processing machine. Given any occurrence, we can quickly assess potential causes and downstream effects. Pearl has further defined an algebra for determining whether causes can be de-confounded, and which variables need to be controlled for in order to effect the de-confounding, given a causal model [20].

**Fig. 5:**
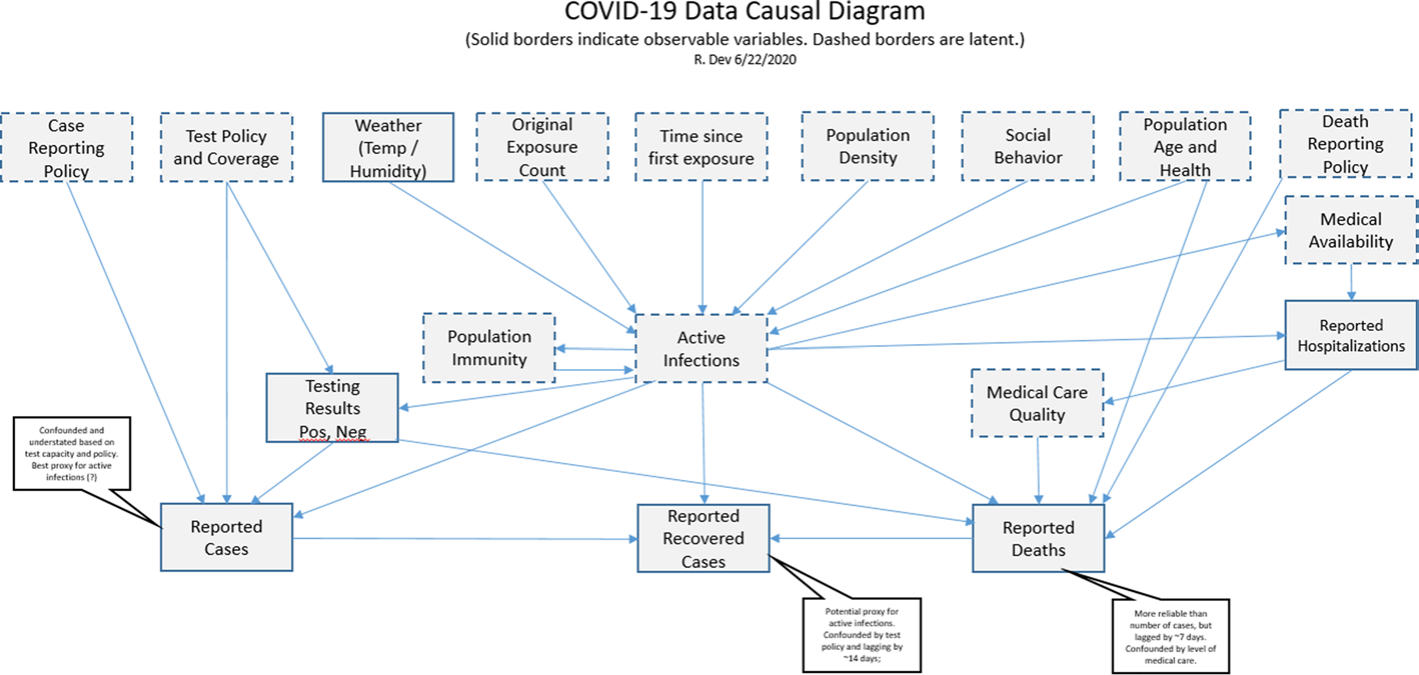
Causal model

Using the model depicted in Fig. 5, we were able to show, for example, that changes in the rate of growth of reported cases is a reasonable proxy for Social Behavior (i.e. Social Distancing). This let us develop the Social Distance Indicator (SDI) metric, which is described in later sections.

### Epidemiological model

The system embeds a classical epidemiological model known as SIR [18]. The SIR model predicts the changes in Susceptibility, Infection, and Recovery using a set of differential equations. This allows us to estimate quantities such as Active Infections, Recovered Infections, Percent Immunity, Time-to-peak, Deaths-at-peak, and Time-to-recovery.

In practice, the SIR model gives us a good estimate of Active versus Recovered infections, but predictive power is limited due to rapidly changing social and societal behaviors. In animal epidemiology, the growth rate (R) is typically identical to the Basic Reproductive Rate (i.e. R0) of the virus. In human society, there are both innate and orchestrated responses to a pandemic that cause R to rapidly diverge from R0. Changes in behavior such as quarantines, social distancing, and enhanced hygiene can quickly dampen the growth rate, whereas returning to normal behavior can rapidly increase the rate. Therefore, any predictions of growth must model the expected changes in human behavior, which is beyond the scope of the current system.

### Data filtering

In the first iteration of the system, we noticed some unexpected swings in R values. Upon analysis, it was discovered that many locations will make corrections to their cumulative case and death data retroactively, either due to changes in reporting policy or correction of previous errors. This sometimes results in cumulative values shrinking, and differential values (e.g. growth) turning negative. Other times, this results in large numbers of cases that occurred previously being dumped into a single day’s data. Both situations can badly distort differential growth calculations. In fact, these adjustments are irrelevant for growth calculations as well as any other calculations that are based on sequential changes (such as the Active Cases calculation) since they are anachronous—not received in the order that they actually occurred. These adjustments, while detrimental to sequential calculations, are important to cumulative values.

We therefore added a Smoothing Filter that calculates an alternate time series for Cases and Deaths with most of the effect of these anachronistic changes removed. This greatly improved the stability and dependability of the sequence dependent metrics, while still allowing use of the original time series for sequence independent metrics. This filter process is described below under Metric Details.

### Infection state

The levels of cR and mR along with some other data, allow us to classify an outbreak according to its stage:

- Spreading—Number of active infections is rapidly increasing (R > = 1.5) and the scale of the infection is probably beyond containment.
- Emerging—Number of active infections is rapidly increasing (R > = 1.5), but is small enough to potentially contain.
- Stabilizing—Infection slowly growing (1.1 < = R < 1.5).
- Stabilized—Number of active infections is approximately stable (0.9 < = R < 1.1).
- Recovering—Number of active infections is shrinking(R < 0.9), but is still beyond containment.
- Recovered—Number of active infections is shrinking or stable, and scale is containable.

These define the potential values of the Infection State at a given location.

### Other metrics

Given a reasonable estimate of R, cR, and mR, we can begin to infer some other metrics that further illuminate the nature of the infection. Metrics are developed to provide insight into the dynamic state of the infection within a location. They may illustrate temporal changes as well as contemporaneous relationships within the data.

 Contagion Risk is the likelihood of meeting at least one infected person during one hundred random encounters.

The Case Fatality Rate (CFR)is the likelihood that someone who tests positive for the virus will die. This is useful for comparing medical conditions between locations with a similar testing and reporting protocol and testing constraints. It is somewhat confounded by changes in testing availability. It almost always overstates the fatality of the infection and should not be confused with the Infection Fatality Rate (IFR).

The Infection Fatality Rate (IFR) is the likelihood that someone who catches the infection will die. This is a very elusive number due to the difficulty in estimating the actual number of infections in a population. This can be retroactively assessed via antibody testing, or approximated through calibrated adjustments.

Cases Per 100 K combines location population data with the Covid-19 reported data to look at the proportion of a population that has tested positive for the virus. This is useful to normalize the infection rates across populations of different sizes. We use “per 100,000” as our scaling factor since it is an easier number to work with than the tiny numbers one would get using a per capita calculation.

Deaths Per 100 K looks at the death rate per 100,000 population at a given location.

Immune Percent identifies the percentage of the population which has recovered from the infection and are presumed to be immune. As a larger proportion of the population becomes immune, the spread of the virus is dampened until at some level so called “herd immunity” is attained. At that point, it is difficult for the infection to continue as there are too few non-immune targets.

The Heat Index is a composite metric that combines a number of relevant metrics to indicate the relative level of attention a given location needs. This index is calibrated such that values greater than 1 indicate that attention is likely needed.

Indicators are a type of metric that can have negative or positive values. We define our indicators such that negative values imply negative outcomes. Indicators highlight both the direction and relative magnitude of change.

The Social Distance Indicator (SDI), based on change in Case Rate (cR), provides insight into the level of social distancing being practiced by a population. All other things being equal, a reduction in R is caused by an increase in social distancing, while an increasing R is indicative of reduced social distancing. This can be somewhat confounded by changes in testing policy and availability, but in practice is a good short-term indicator.

The Medical Indicator (MDI) is based on changes in the ratio of Case Rate (cR) to Mortality Rate (mR). If all else is held constant, this ratio would settle at a consistent value, as the rate of increase in deaths would be proportional to the rate of growth in cases. Therefore, a decrease in the ratio signals that something has changed for the worse. In practice, this can be caused by a number of factors: (1) Testing is not growing as fast as the infection, (2) Medical Care is worsening or (3) Rapid changes in R combined with the time lag of deaths can cause skew between the two. If we adjust for the time lag, then either of the first two causes can be considered medical care issues. Thus decreases in this ratio will result in a negative Medical Indicator.

The Short-Term Indicator (STI) is a predictive indicator that attempts to determine if the infection is likely to get worse (negative values) or get better within a few days.

The Early Warning Indicator (EWI) predicts major shifts (inflection points) in the momentum of the infection. It is meaningful when an infection is moving from a neutral or recovering state to a spreading state. It is also meaningful when an infection is transitioning from growth to stability.

### Surge detection

The system tracks ebbs and flows in the infection rate to show multiple “surges” or “waves” of infection. We define a surge as a transition from a shrinking (R < 0.9) state to a growing state (R > = 1.1). We track the start dates, peaks, and durations of each surge. Knowing the surge number and start date helps in understanding the oscillations that a location goes through over the life of the infection.

### Commentary

The metrics above paint a fairly clear picture of the state of the infection in any location at any given time. Interpreting them, however, requires a detailed understanding of the meaning of each metric and the range of values that it can assume.

We therefore created an interpretive commentary that describes the state of the infection for each location. This commentary combines the various metrics with expert qualitative assessment to form as complete a picture as possible, depending on the Infection State. For example:

As of Aug 20, 2020, US-FLORIDA has improved to a Recovering state from a previous state of Stabilized. The infection is slowly decreasing (R = 0.81). There are currently 49,270 active cases. New Cases are currently 30,750 per week, down 62% from a peak of 79,920 per week. New Deaths are currently 1,035 per week, down 0% from a peak of 1,035 per week. This is the 4th surge in infections, which started on the week of May 29, 2020. With 1,035 new deaths, this is the worst week so far for deaths during this surge. The Contagion Risk is very high at 49.9%. This is the likelihood of meeting an infected person during one hundred random encounters. It appears that the level of social distancing has increased significantly, resulting in lower levels of infection growth. The Case Fatality Rate (CFR) is estimated as 1.7%. This is much lower than the average CFR of 3.6%. Preliminary estimates suggest that 7% of the population may have been infected and are presumed immune. This is not enough to significantly slow the spread of the virus. This preliminary estimation also implies an Infection Fatality Rate (IFR) of roughly 0.6%. The Short-Term Indicator (STI) suggests that the infection is likely to slow somewhat over the next few days.

The commentary consists of several sections, each centered on the interpretation of one or more metrics:

- Infection State and Previous state if changed.
- Number of active cases and implications.
- Surge information.
- Contagion Risk and qualitative assessment.
- Social Distancing assessment.
- Medical Conditions.
- Case Fatality Rate.
- Immune Percentage.
- Hot Spots information (if on Hot Spots list).
- Predictive Indicators..

### Metrics details

Proposed metrics are based on the following definitions:

- Constants .
  - Infection Period(IP)—The average length of time during which an individual remains infections. This is currently set to 10 days.
  - Infection Case Ratio(ICR)—The average ratio of Actual infections to cases. This is a gross estimate of the ratio of all infections (Asymptomatic, Subclinical, Clinical) to Confirmed Cases. Although this is treated as a constant for rough estimation, it is known that this number varies over time as well as location, based on testing availability. This is currently set to 3.0 based on estimates by Penn State [2].
  - Metric Window (MW)—The number of days over which growth metrics are calculated. This is currently set to 7.
  - minActiveThreshold—The minimum fraction of the population with active infections in a location to be considered beyond containment. This is currently set to 0.0003.
  - hiScaleFactor—A scaling factor for Heat Index that provides a threshold for the Hot Spots list. This is calibrated such that Heat Index > = 1.0 identifies locations requiring attention. This is currently set to 5.0.
- Input Statistics .
  - Cases—Cumulative cases for a given location.
  - Deaths—Cumulative deaths for a given location.
  - Hospitalizations—Cumulative hospitalizations for a given location.
  - Positive—Cumulative number of positive tests for a given location.
  - Negative—Cumulative number of negative tests for a given location.
  - Population—The number of individuals living in a given location.

### Adjusted cases and deaths

Various locations will occasionally produce anachronous data. That is, data that is not arriving in correct time sequence. This typically occurs when there is a change in reporting policy for the location, or when errors were found in the reporting process and corrections are applied retroactively. In these cases, it is common for large batches of cases or deaths to be suddenly dumped into a single days reporting. Likewise, downward corrections are occasionally seen, that can cause the cumulative values to become non monotonic. These occurrences can dramatically distort resulting metrics, especially those that depend on the difference in cumulative totals among periods, such as growth rate computations. To compensate for this, we subject the source data to a smoothing filter. This produces a set of alternate inputs that have removed these spikes and reversals. These alternate values can then be used to calculate more consistent differential values.

This filter is applied to the incoming data, both Cases and Deaths. It limits any daily change to the MW-day moving MIN of the series and 1.25 * the MW-day moving MAX of the series.

It then reconstructs a new adjusted time series based on these restricted changes. A change greater than 1.25 from day to day implies a growth rate(R) greater than 10, which is larger than any expected maximum real growth rate, At the same time, the filter removes any negative changes, by bounding the newCases and newDeaths to greater than or equal to zero.

- newCases(T) = MAX(Cases(T) - Cases(T-1), 0).
- casesMax(T) = MAX(newCases(T-MW − 1), newCases(T-MW), … ,newCases(T-1)).
- casesMin(T) = MIN(newCases(T-MW − 1), newCases(T-MW), … ,newCases(T-1)).
- adjustedNewCases(T) = IF(newCases(T) > 1.25 * casesMax(T), 1.25 * casesMax(T), IF(newCases(T) < casesMin(T) / 1.25, casesMin / 1.25, newCases(T))).
- adjustedCases(T) = adjustedNewCases(1) + adjustedNewCases(2) + … adjustedNewCases(T).
- newDeaths(T) = MAX(Deaths(T) - Deaths(T-1), 0).
- deathsMax(T) = MAX(newDeaths(T-MW − 1), new Deaths (T-MW), … ,newDeaths(T-1)).
- deathsMin(T) = MIN(newDeaths (T-MW − 1), newDeaths (T-MW), … ,newDeaths (T-1)).
- adjustedNewDeaths (T) = IF(newDeaths(T) > 1.25 * deathsMax(T), 1.25 * deathsMax(T), IF(newDeaths(T) < deathsMin(T) / 1.25, deathsMin / 1.25, newDeaths(T))).
- adjustedDeaths(T) = adjustedNewDeaths(1) + adjustedNewDeaths(2) + … adjustedNewDeaths(T)..

### Metrices

These are calculated based on an MW (e.g. 7) day sliding window. T refers to the current day, while T-MW refers to MW days previous. Note: please see the definition of adjCase from section Adjusted Cases and Deaths.

- cR—The effective case growth rate.
- cR = ((adjustedCases(T)– adjustedCases(T-MW)) / (adjustedCases(T-MW)-adjustedCases(T-2 MW)))^(IP/MW).
- mR—The effective mortality growth rate.
  mR = ((adjustedDeaths(T)– adjustedDeaths(T-MW)) − (adjustedDeaths(T-MW) − adjustedDeaths(T-2 MW)))^(IP/MW).
- R—Estimate of the effective reproductive rate. This is based on a geometric mean of cR and mR. Some constraints are placed on the values to reduce the effect of very noisy data.$R = \sqrt{\left(MIN\right(cR, mR + 1.0\left) * MIN\right(mR, cR + 1.0\left)\right)}$
- Active—The estimated number of active (i.e. infectious) cases.
  Active = adjustedCases(T) - adjustedCases(T-IP).
- Recovered—The number of cases that are considered recovered.
  Recovered = Cases - Active - Deaths.
- ContagionRisk—The likelihood of encountering at least one infected person during 100 random encounters.
  $$Contagion\,Risk = 1 - \left( {1 - \left( {{{Active} \mathord{\left/ {\vphantom {{Active} {Population}}} \right. \kern-\nulldelimiterspace} {Population}}} \right)} \right)^{{100}}$$
- Case Fatality Rate (CFR)—The likelihood of dying given a positive test result.
  $${\text{CFR = }}{{{\text{Adjusted Cases }}\left( {{\text{T}} - {\text{IP}}} \right)} \mathord{\left/ {\vphantom {{{\text{Adjusted Cases }}\left( {{\text{T}} - {\text{IP}}} \right)} {{\text{adjusted Deaths (T)}}}}} \right. \kern-\nulldelimiterspace} {{\text{adjusted Deaths (T)}}}}$$
- Infection Fatality Rate (IFR)—The likelihood of dying, having acquired an infection. This is a gross approximation assuming a constant ICR.
  IFR = CFR * ICR.
- immunePct—The fraction of the population that has recovered from the infection and are considered immune:
  $$Immune\,Pct = {{Recovered*ICR} \mathord{\left/ {\vphantom {{Recovered*ICR} {Population}}} \right. \kern-\nulldelimiterspace} {Population}}.$$
- Infection State (IState)—A qualitative metric that models the state of the infection. It will assign one of the following states to the infection within a location:
  1) INITIAL, 2)RECOVERED, 3) RECOVERING, 4) STABILIZED, 5) STABILIZING, 6) EMERGING, 7) SPREADING. These are assigned based on a series of cascading predicate tests. The first true predicate assigns the state.
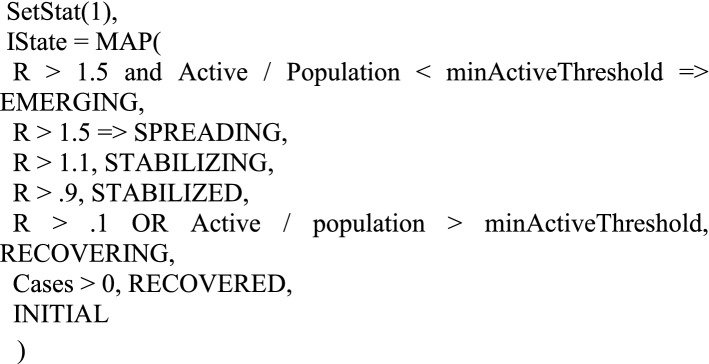
- HeatIndex(HI)—A composite metric that takes into account scale, growth rate, social distancing, medical conditions, and Contagion Risk. This metric is scaled such that values > = 1.0 are considered Hot Spots needing attention.
  $$Heat\,Index = {{LOG(Active)*(MIN(cR,mR + 1) + MIN(mR,cR + 1) + MI + SDI + ContagionRisk)} \mathord{\left/ {\vphantom {{LOG(Active)*(MIN(cR,mR + 1) + MIN(mR,cR + 1) + MI + SDI + ContagionRisk)} {hiScaleFactor}}} \right. \kern-\nulldelimiterspace} {hiScaleFactor}}$$

### Indicators

Indicators are zero-based, with negative numbers indicating negative outcomes, and positive numbers positive outcomes.

- Social Distance Indicator (SDI)—Based on the ratio of the current cR to the previous cR. dcR = cR(T) / cR(T-MW).
  $$SDI = IF(dcR > 1,1 - dcR,1/dcR - 1).$$
- Medical Indicator (MI)—Based on the ratio of case growth (cR) to mortality growth (mR).
  $$cmRatio = {{cR} \mathord{\left/ {\vphantom {{cR} {mR}}} \right. \kern-\nulldelimiterspace} {mR}}.$$
  $$MI = IF\left( {{{cmRatio > 1,cmRatio - 1,1 - 1} \mathord{\left/ {\vphantom {{cmRatio > 1,cmRatio - 1,1 - 1} {cmRatio}}} \right. \kern-\nulldelimiterspace} {cmRatio}}} \right).$$
- Short-term Indicator (STI)—A short term directional predictor (period 2–3 days).
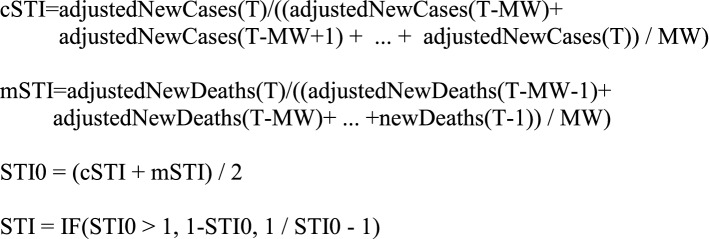
- Early Warning Indicator (EWI)—EWI is a pseudo-predictor. It uses predictable changes in the ratio of newCases to newDeaths to detect major inflections. It generates a positive signal when R (as computed above) is likely to transition from above one to below one within one to two weeks. It generates a negative signal in advance of an R transition from below one to greater than one. It is not a true predictor in that it detects that the inflection has already occurred, but did not show up in the computed R because of its lagging mR component.
  $$EWI0 = SDI - MI.$$
  $$EWI = IF\left( {SDI < - 0.2ANDMI > 0.2,EWI0,IF\left( {SDI > 0.2ANDMI < - 0.2,EWI0,0} \right)} \right).$$

### Data pipeline

Our data pipeline is a One-Stop-Shop scalable solution from seamless data collecting, ingestion, ETL, analytics to governing and monitoring built on top of HPCC Systems. Each component of the pipeline runs, as a job in the HPCC Systems cluster. These jobs are scheduled to run automatically once the new incoming data is received without human intervention. Below is an introduction of each component of the pipeline.

Collection: In our pipeline, the source data is automatically collected by monitoring and pulling the new data from data source and automatically uploaded to the HPCC Systems cluster for Data Ingestion.

Ingestion: once the data are collected and automatically uploaded for ingestion, the ingestor will automatically search the newly uploaded files, and perform the ingestion process into the ETL system.

ETL: By transforming, enhancing and cleaning the data, the processed data including Covid19 metrics are stored in the HPCC Systems Data Lake so that data scientists and researchers can apply data analytics to extract useful information.

Analysis: As introduced in the Metrics section, SIR model and Covid-19 indicators are developed in the HPCC System for Covid19 analysis and prediction. The built-in Machine Learning Library of the HPCC Systems is a great tool for data scientist and researcher to conduct data analytics and statistic inference as well. It includes, but is not limited to, regression bundles, classification bundles, clustering bundles and Deep Learning bundle as well.

## Data monitoring

**Fig. 6:**
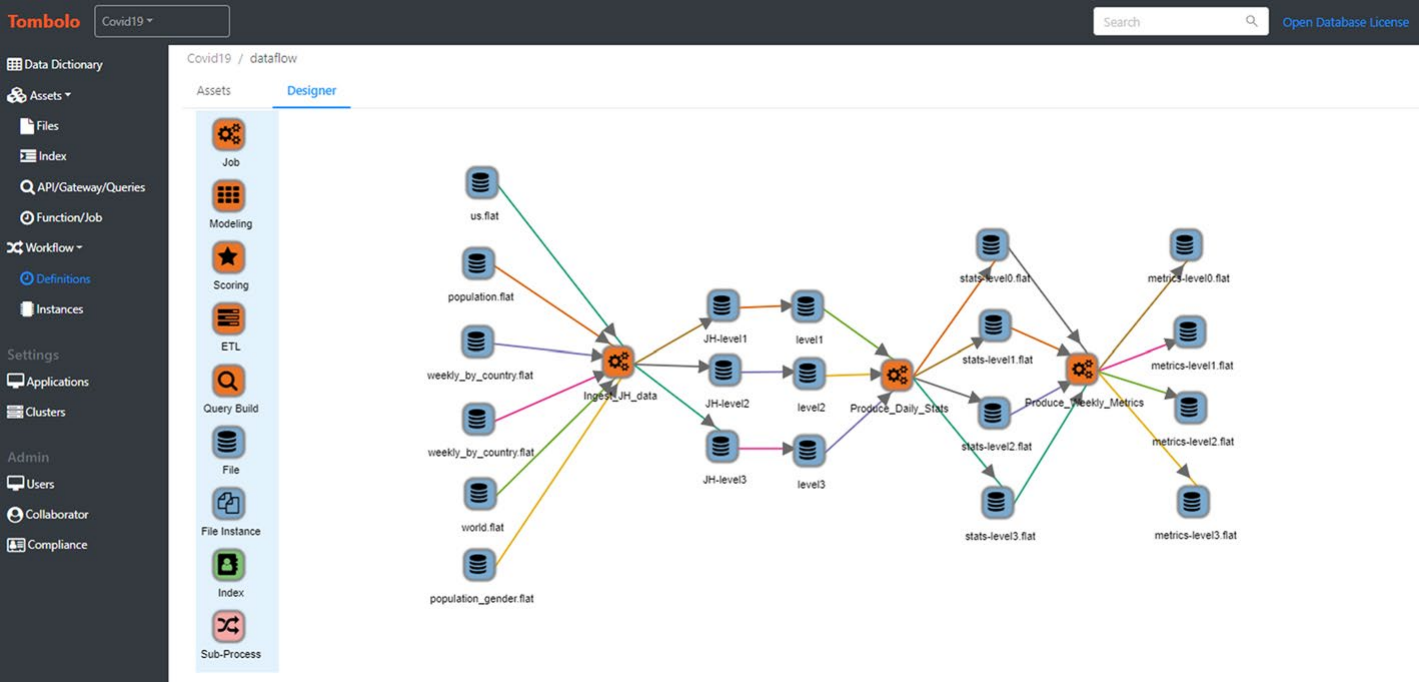
Tombolo Covid-19 Workflow

Data monitoring is managed by Tombolo, HPCC Systems Data Catalog tool. Whenever a job fails, Tombolo will instantly identify the failure and automatically send an email notification to the system administrator for the failure. For data governing, the pipeline on HPCC Systems is defined as workflow in Tombolo, as shown in Fig. 6. Each run of the workflow is an instance and the status of each job is recorded. If any job failed, it will automatically send an email notification to the administrator.

The main data sources are John Hopkins University (daily cases and deaths), US Census Bureau (US population), and UN DESA (World population). The lake data and the workflow can be viewed using HPCC Systems Data Catalog tool Tombolo (version 0.5).

The system is available at: https://tombolo.hpccsystems.com.

Log in information: User name: CovidTracker Password: HPCCSystems.

A node in the workflow can be selected and double clicked to view the details. The following is an example of the details of a metrics file (Fig. 6).

## HPCC systems COVID-19 tracker results

With a user-friendly interface, an Automatic Big Data Pipeline as powerful data solution and the model and metrics as pandemic indicators, the proposed work is available as a public Website at https://covid19.hpccsystems.com.

The Smoothing Filter described above has done a good job of eliminating anachronous data dumps, while having little impact on the natural time series. If differential values such as R were computed on the raw time series, severe distortions would result.

**Fig. 7:**
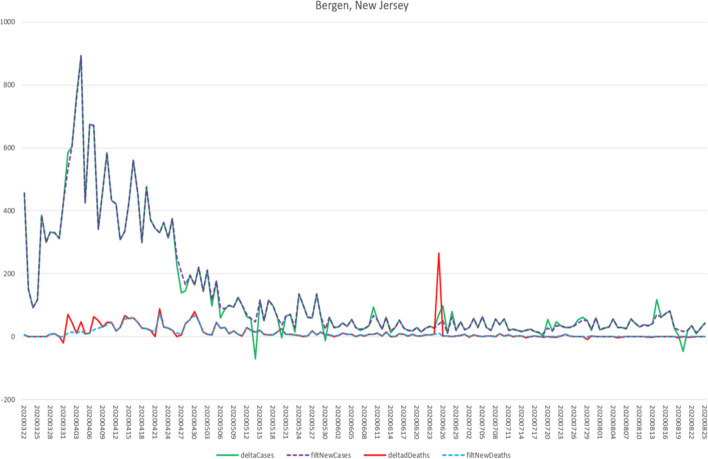
Smoothing filter—County level

**Fig. 8:**
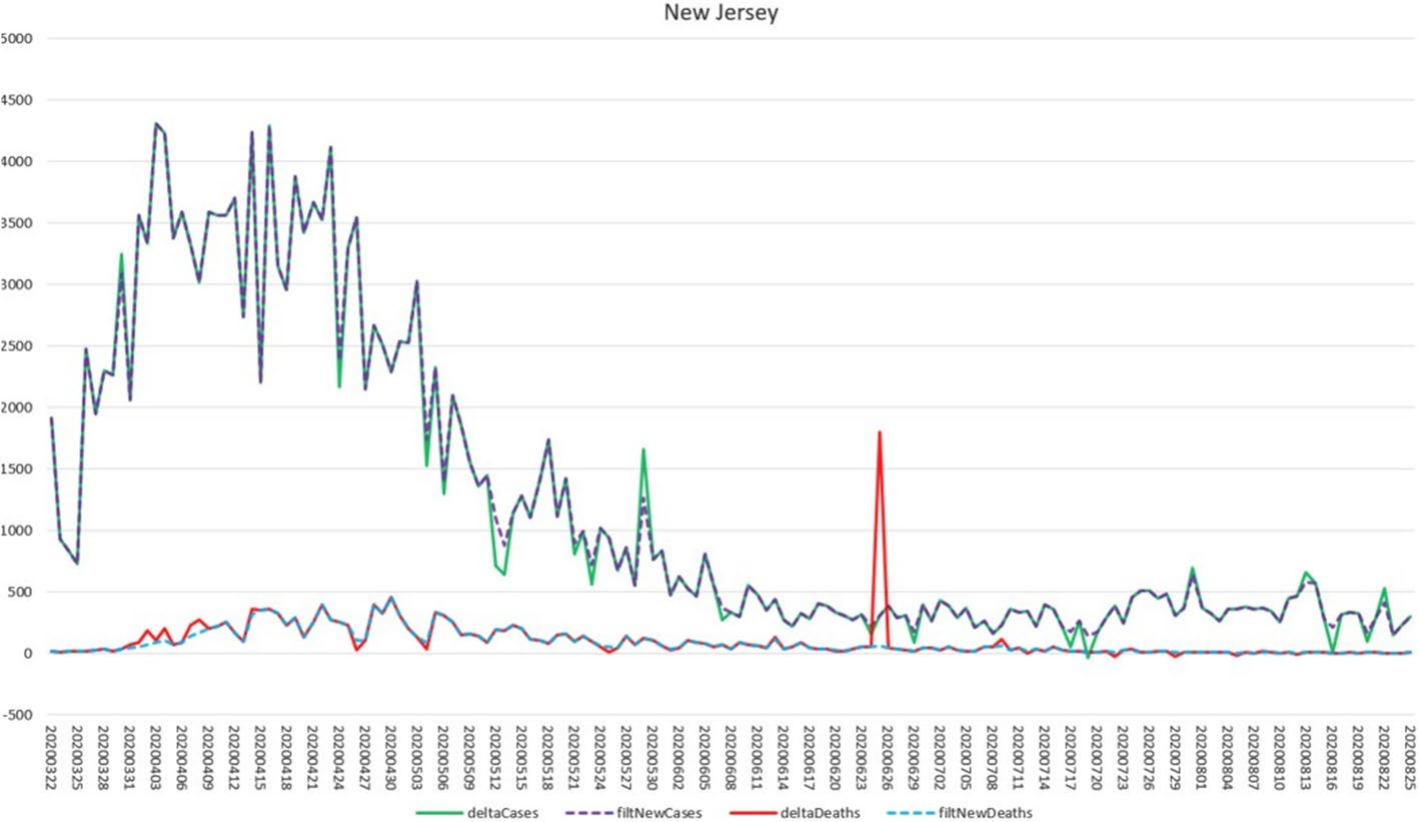
Smoothing filter—State level. The chart illustrates the filter operating at a higher level (the state of New Jersey). Note that statewide the Deaths adjustment at 06/25 indicates nearly 1800 one day deaths compared to an average of around 50. This is effectively filtered out along with several anomalously high and low spikes

The charts in Figs. 7 and 8 show the original time series data as well as the filtered results at two levels—the county of Bergen, New Jersey, USA, and the state of New Jersey. “deltaCases” is the difference in Cases from period T-1 to period T. “deltaDeaths” is the equivalent for Deaths. “filtNewCases” and “filtNewDeaths” are the adjusted versions of the delta time series based on the Smoothing Filter.

Note that the anachronous spike in deaths (red line), and the negative spikes in delta cases (green line) have been effectively removed from the filtered series. Also note that toward the end of the green line several anomalous spikes have also been attenuated.

A unique aspect of this system is the ability to produce a daily English commentary reflecting the state of each location. The commentary combines metrics-based inferences with enough background information to help the reader understand the implications. For example, here is a commentary describing the state of the world-wide infection for June 18, 2020.

“The World has worsened to a Stabilizing state from a previous state of Stabilized. The infection is slowly increasing (R = 1.18). At this growth rate, new infections and deaths will double every 42 days. This is the 2nd surge in infections, which started on the week of May 28, 2020. With 989,711 new cases and 32,758 new deaths, this is the worst week yet for cases and deaths during this surge. It appears that the level of social distancing is decreasing, which may result in higher levels of infection growth. The Case Fatality Rate (CFR) is estimated as 6.4 %. The Short-Term Indicator suggests that the infection is likely to worsen over the course of the next few days.“

### User interface

**Fig. 9:**
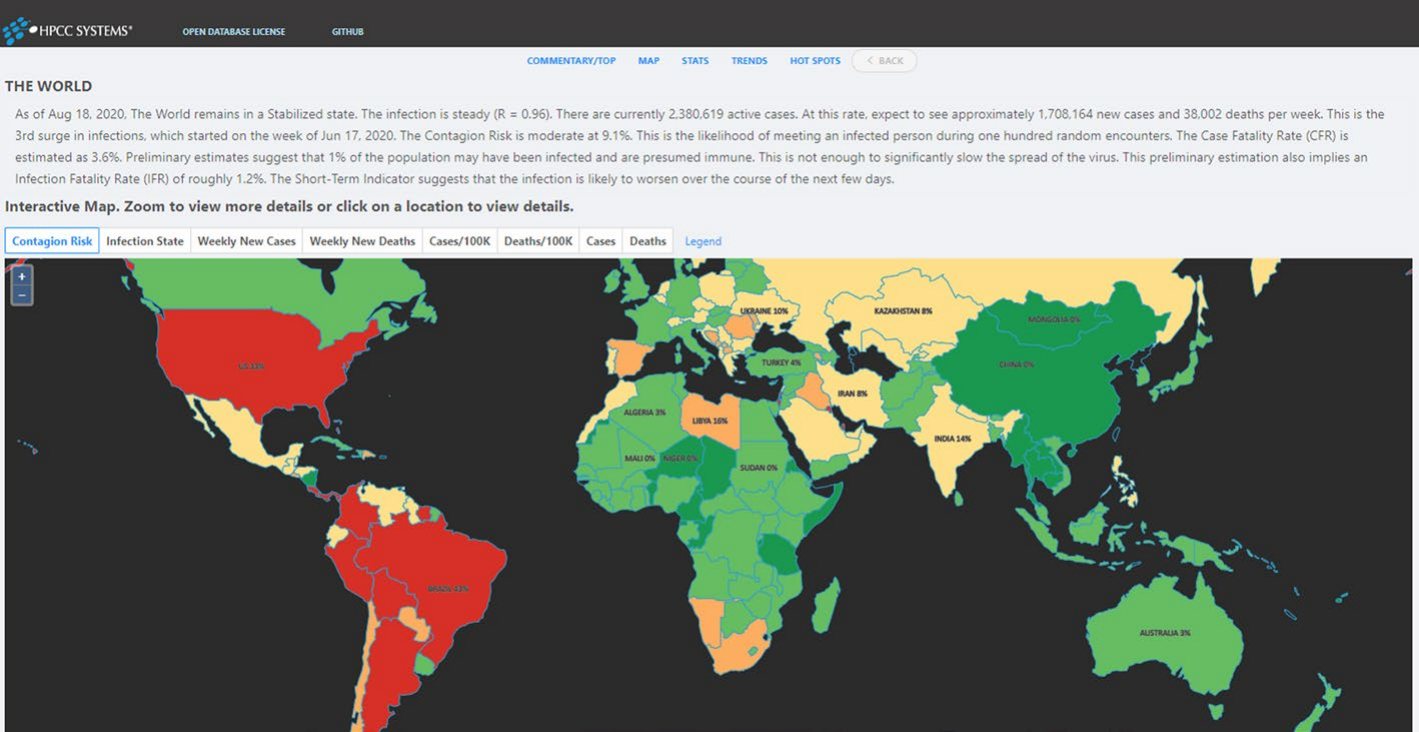
World Map View: shows aspects of the infection through color coding on a map. The map can be color-coded by a number of attributes including Infection State, New Cases, New Deaths, Cases per 100 K, Deaths per 100 K, Total Cases and Total Deaths

The system provides a user-friendly web-based interface for viewing COVID-19 data and metrics. World, Country, and Regional maps are color-coded to represent any of various selectable attributes of the infection at those locations. Clicking on any given location brings up a set of pages that provides details about that location—from raw statistics to charts to advanced metrics and commentary. The user interface provides several ways to navigate such as Map View, Trend View, Stats View, Hotspots View and so on. Below is detailed introduction of each view. The Website is released to the public at http://covid19.hpccsystem.com (Fig. 9).

*Map View* shows aspects of the infection through color coding on a map. The map can be color coded by a number of attributes including Infection State, New Cases, New Deaths, Cases per 100 K, Deaths per 100 K, Total Cases and Total Deaths. On Map View, users can drilldown to the lowest location as possible. Currently supported Views include World View, Country View, Province/State View, City/County View.

**Fig. 10:**
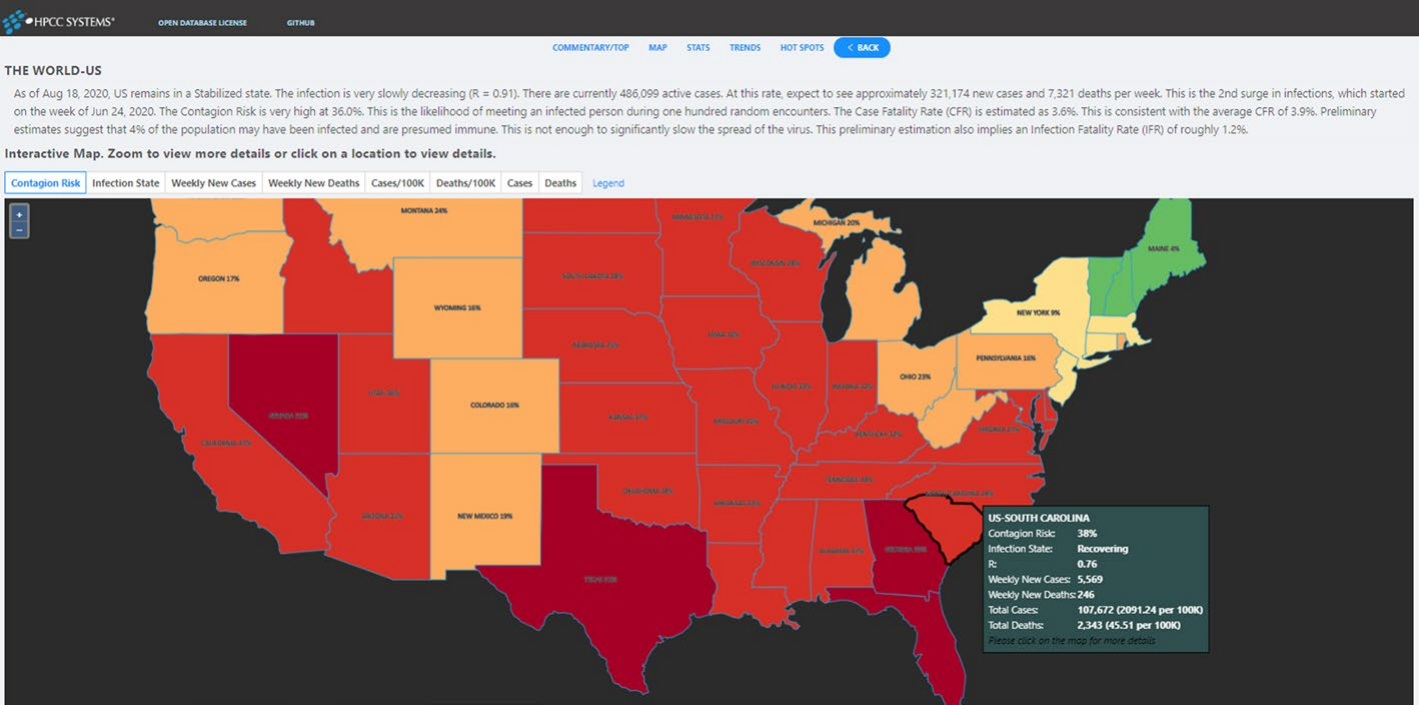
US Country Map View

Map View displays data in two perspectives: (i) By default the immediate state of the infection (current week), and (ii) Use the navigation buttons to check the infection state in the past weeks. It can also automatically animate all the historical Map Views one by one by clicking the auto-play button (Fig. 10).

**Fig. 11:**
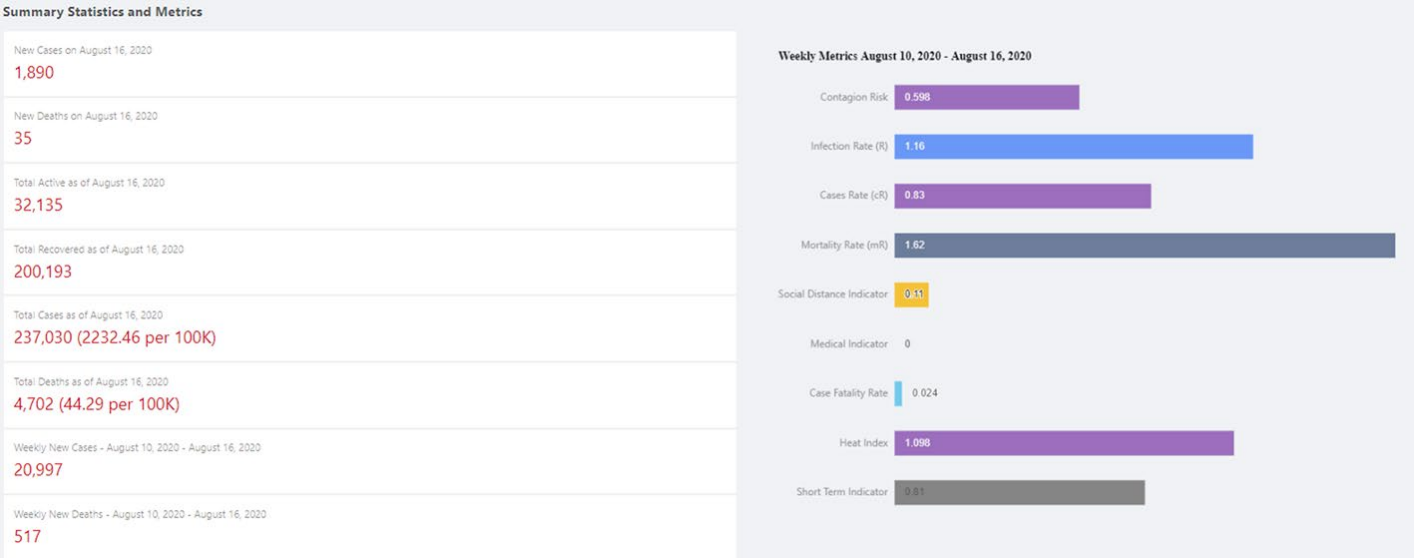
Stats View: summary statistics and metrics at each location

Stats View shows the summary statistics and metrics at each location, as illustrated in Fig. 11. The location can be at the world level, country level, province/state level or city/country level. The statistics include, but are not limited to, daily new cases, daily new deaths, cumulative cases and cumulative deaths. It also includes all the metrics introduced in the previous section. The metrics are displayed in a bar chart.

**Fig. 12:**
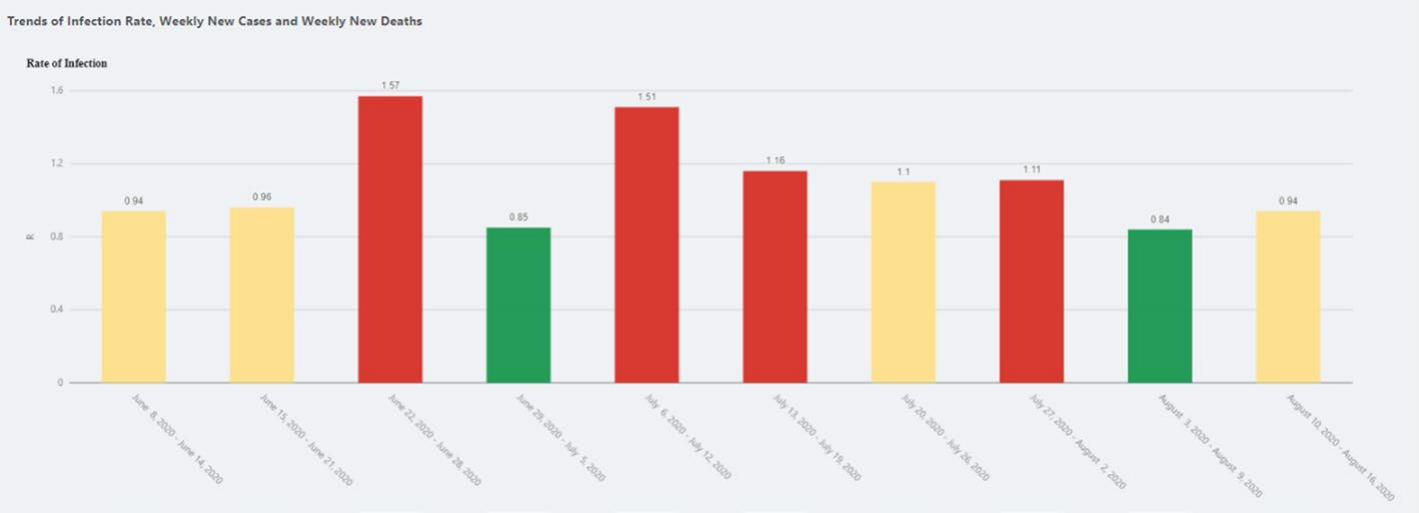
Trend View—Infection rate

**Fig. 13:**
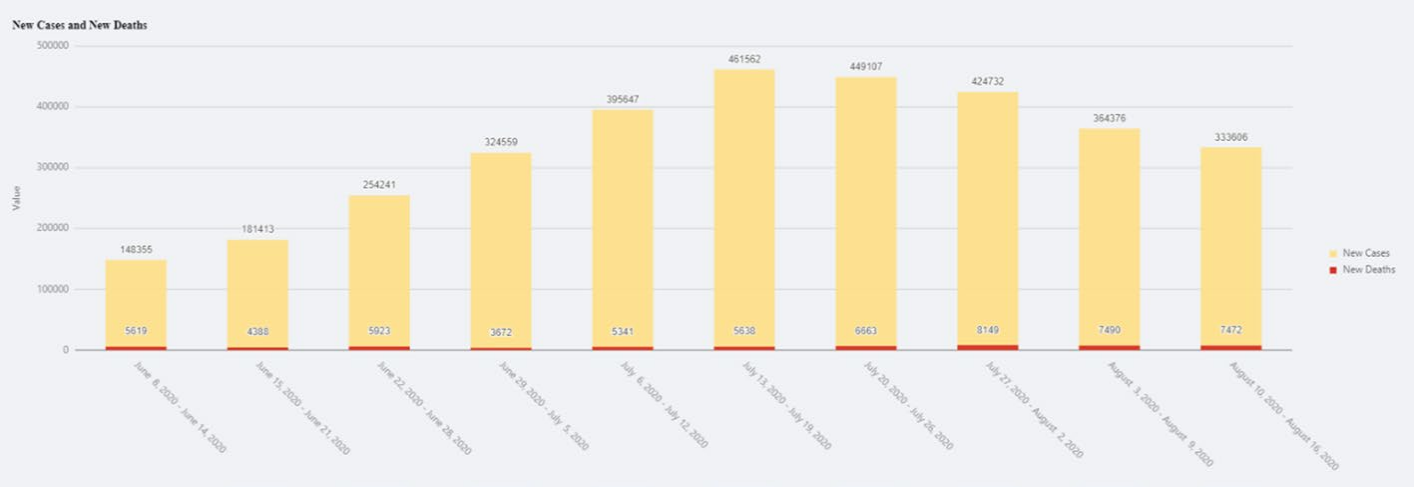
Trend View—New cases and new deaths

Trend View shows the trend of infection rate, weekly new cases and weekly new deaths, as shown in Figs. 12 and 13. The details of the definitions can be found in the previous section.

**Fig. 14:**
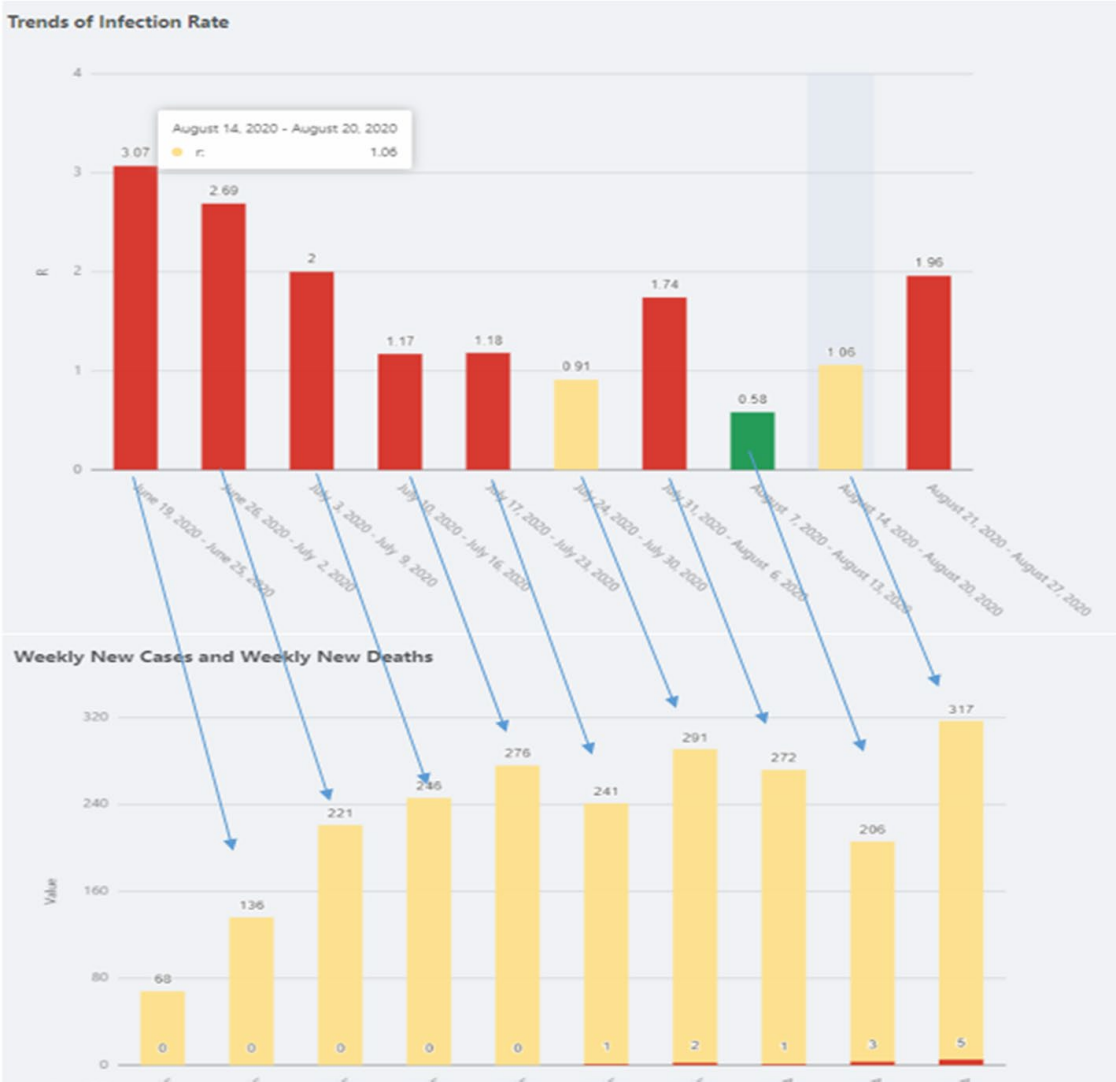
Weekly comparison between Infection Rate (IR) Trend (image on the top) to new Cases Trend (at the bottom) of Clarke County, Georgia in the past ten weeks

Except for the useful commentary and summary statistics, the indicators represent another great tool for COVID-19 trend prediction indifferent communities. Figure 14 shows an example in Clarke County, Georgia.

As we can see from Fig. 14, when IR shows spreading (IR > 1.0) for at least two consecutive weeks, it is likely that in the following weeks there will be a jump of new cases and the trend will keep going if no action or policy is taken. With this prediction in mind, we could reduce going out or visiting friends to lower the risk of getting infected.

## Conclusions

The described industry research activities have a great potential to advance knowledge within the proposed field of research as well as across different fields, such as medical, healthcare, and public applications. The project helped to build a coalition between FAU and LexisNexis to jointly address public health problems of national and global significance using the state of the art in computer science, big data analytics, data visualization techniques, and decision support systems. The proposed methodology, the including the coalition-building effort will support solutions for a wide range of other public health issues.

The COVID-19 indicator can be used to predict the future trend of COVID-19, but it has its own limitations and cannot explain other factors that can affect the trend such as mobility, weather and others. Our future work will build a stronger model to predict the trend with comprehensive features.
